# Warming reduces fungal endophyte dependency for establishment but amplifies stress-response gene expression in the Antarctic invader *Poa annua*

**DOI:** 10.3389/ffunb.2026.1868127

**Published:** 2026-07-08

**Authors:** Sebastián A. Reyes, Cristian Atala, Cristian Torres-Díaz, Marco A. Molina–Montenegro

**Affiliations:** 1 Centro de Ecología Integrativa, Instituto de Ciencias Biológicas, Universidad de Talca, Talca, Chile; 2 Programa de Doctorado en Ciencias, mención Biología Vegetal y Biotecnología, Instituto de Ciencias Biológicas, Universidad de Talca, Talca, Chile; 3 Laboratorio de Anatomía y Ecología Funcional de Plantas (AEF), Instituto de Biología, Facultad de Ciencias, Pontificia Universidad Católica de Valparaíso, Valparaíso, Chile; 4 Grupo de Investigación en Biodiversidad & Cambio Global (GIBCG), Departamento de Ciencias Básicas, Universidad del Bío-Bío, Chillán, Chile; 5 Centro de Investigación en Estudios Avanzados del Maule (CIEAM), Universidad Católica del Maule, Talca, Chile

**Keywords:** biological invasions, climate change, extreme environments, polar ecosystems, thermotolerance

## Abstract

Fungal endophytes transmitted through seeds are recognized as key drivers of plant invasion success, yet whether their role persists or shifts under climate warming remains poorly understood. Here we address this question in *Poa annua*, the only non-native vascular plant with established self-sustaining populations in Maritime Antarctica, using a factorial experiment on King George Island. Combining open-top chamber warming (OTC^+^) and endophyte manipulation (E^+^ vs. E^−^), we assessed germination, seedling survival, and expression of two stress-response genes (*HSP* and *LEA1*) in this invasive grass. Germination was unaffected by either treatment, while survival was independently improved by both endophyte presence and warming, but their effects did not interact, suggesting that thermal amelioration may reduce reliance on endophyte-mediated buffering for establishment. Under warming, however, endophytes strongly amplified *HSP* expression through a significant OTC × E interaction, indicating conditional molecular priming of the heat-stress response. *LEA1* expression showed the additive effects of both factors. These results suggest that as Antarctic temperatures rise, *P. annua* may progressively decouple from its seed-borne fungal endophytes for establishment while retaining endophyte-amplified transcriptional responses to thermal and hydric stress.

## Introduction

Antarctica is one of the most isolated terrestrial ecosystems on Earth, yet it faces growing pressure from biological invasions as climate warming accelerates glacial retreat and exposes new ice-free substrates that can support vascular plant colonization (Frenot et al., 2005; Convey and Peck, 2019; Rudak et al., 2026). Moreover, warming on the Antarctic Peninsula has been among the most rapid globally (González-Herrero et al., 2022). Biological invasions are a primary threat to the ecological integrity of pristine ecosystems worldwide (Pyšek et al., 2020), and in Antarctica, this threat is compounded by two converging pressures: increasing human activity, which elevates propagule pressure through the unintentional transport of plant propagules, and climate warming, which expands the area of climatically suitable terrain available for colonization (Chown et al., 2012; Convey and Peck, 2019). Milder conditions independently lower the establishment threshold for non-native plants previously excluded by cold, increasing the risk of permanent colonization in newly ice-free areas.

Among the non-native plants documented in Antarctica, *Poa annua* L. (annual bluegrass; Poaceae) is an invasive vascular species first recorded on King George Island in the mid-1980s and subsequently detected at additional sites along the Antarctic Peninsula (Molina-Montenegro et al., 2012; Chwedorzewska et al., 2015). Its establishment success varies with substrate type, microclimate, and distance to propagule sources, with germination rates remaining high across populations from contrasting climatic zones, including polar conditions (Rudak et al., 2018). Invasion risk from this species is not constrained to a single propagule source, and may intensify under ongoing warming as climatically suitable terrain expands (Rudak et al., 2026). Seed-borne fungal endophytes that are maternally transmitted between generations have emerged as a key factor underpinning its invasive success (Ballesteros et al., 2022). These microorganisms increase seedling germination, biomass accumulation, and survival in Antarctic soils, while promoting the release of allelochemicals that suppress native plant growth (Molina-Montenegro et al., 2023).

At the molecular level, fungal endophytes are known to modulate the expression of stress-protective genes in plant invaders (Acuña-Rodríguez et al., 2024), including heat shock proteins (HSPs). These molecular chaperones stabilize proteins under thermal stress and confer acquired thermotolerance (Kotak et al., 2007; Waters, 2012). In addition, late embryogenesis abundant (LEA) proteins protect cellular structures from desiccation and osmotic damage (Hundertmark and Hincha, 2008). Although HSPs are classically associated with thermal protection from overheating, they are also relevant under the moderate warming generated by open-top chamber (OTC) experiments, which reliably elevate air and soil temperatures by 1–3 °C above ambient during the growing season in polar ecosystems, mimicking near-future climate scenarios (Bokhorst et al., 2013; Newsham et al., 2022). Similarly, LEA proteins respond not only to desiccation but also to the osmotic stress associated with freeze-thaw cycles and episodic drought on newly exposed Antarctic substrates (Acuña-Rodríguez et al., 2024). Whether endophytes regulate these responses specifically in *P. annua* under simulated Antarctic warming has not been examined.

Studies on Antarctic native plants have shown that fungal endophyte-mediated benefits on survival and ecophysiological performance are context-dependent: positive under current stress conditions but may be neutral or attenuated under projected warmer scenarios (Torres-Díaz et al., 2016; Hereme et al., 2020). Whether this applies to *P. annua* in Antarctica —and whether endophytes modulate not only competitive outcomes, but also the transcriptional stress response of this plant under warming— remains an open question. Understanding these dynamics is critical because temperature projections for Maritime Antarctica anticipate a continued warming and an expansion of ice-free terrain that will alter the establishment for invasive species (Convey and Peck, 2019). The molecular priming of stress responses by endophytes could represent an additional layer of invasive advantage that has been largely overlooked.

Here we report the effects of passive warming via open-top chambers (OTCs) and fungal endophyte presence on seedling germination, survival, and expression of *HSP* and *LEA1* stress-response genes in *P. annua* grown in Antarctica. We hypothesized that (i) endophytes improve seedling survival under current ambient conditions but that their benefit diminishes under warming as thermal barriers to establishment are reduced; and (ii) endophytes amplify the transcriptional stress response (particularly of *HSP* and *LEA1* gene expression) under warming, priming transcriptional responses in *P. annua* associated with thermal and drought stress. Our results reveal a context-dependent role of fungal endophytes in the establishment of this invasive grass, with implications for predicting its invasive dynamics as Antarctic warming continues.

## Materials and methods

### Plant sampling and experimental design

**Figure 1:**
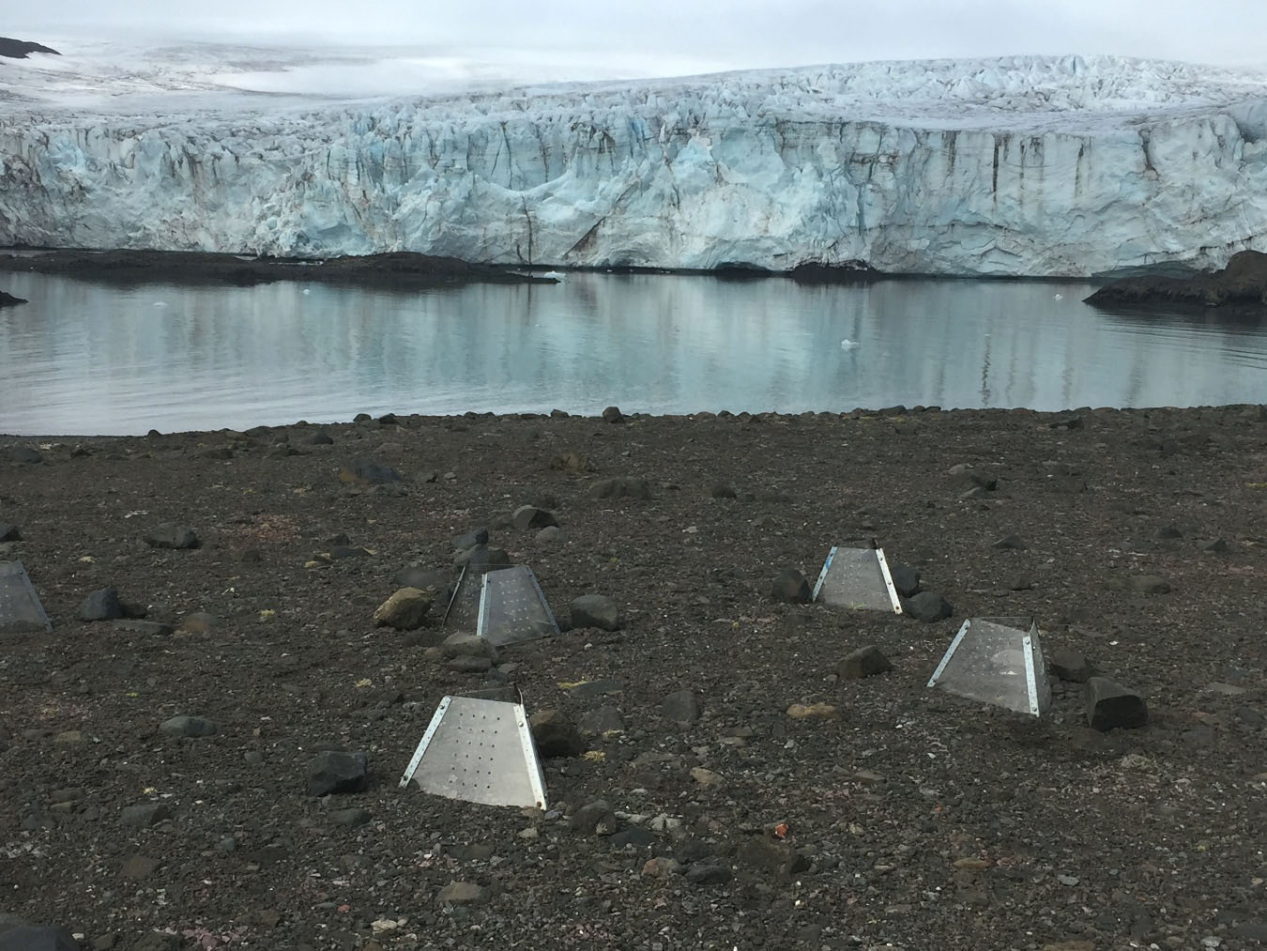
Experimental site on the forefield of the Ecology Glacier, King George Island, South Shetland Islands (Maritime Antarctica). Open-top chambers (OTCs) installed on bare rocky substrate are visible in the foreground, with the glacier front and proglacial lake in the background.

Field work was carried out near the H. Arctowski Polish Antarctic Station on the western shore of Admiralty Bay, King George Island, South Shetland Islands (Maritime Antarctica). This coastal location, situated at sea level on the western shore of Admiralty Bay in proximity to both the ocean and the Ecology Glacier, is characterized by persistently high relative air humidity throughout the austral summer (Plenzler et al., 2026). Seeds and tillers of *Poa annua* were collected in January 2017 during the 53rd Chilean Antarctic Scientific Expedition (ECA-53) from plants growing on the forefield of the Ecology Glacier (62.169°S, 58.472°W; **Figure 1**), a site that has been monitored as a reference location for *P. annua* invasion dynamics in Maritime Antarctica.

Field experiments to assess the effects of warming and fungal endophytes on plants were conducted in two ways. First, 140 and forty tillers, originated from seeds collected from 14 P*. annua* plants inhabiting the vicinity of the Ecology Glacier were allocated to four treatments. Of the 140 tillers, 70 were reduced in its fungal endophytes (E^-^ treatment) and the other 70 plants maintained its fungal endophytes (E^+^ treatment). All individuals were planted individually in paper pulp pots (50 ml capacity) containing sterilized soil from the Ecology Glacier forefield. Then, 10 pots, five containing *P. annua* with reduced fungal endophytes and five containing intact fungal endophytes, were sunk into pits inside each of the seven OTCs, with the lips of the pots at the same level as the soil surface. A further 10 pots, again consisting of five pots containing *P. annua* with reduced fungal endophytes and five pots containing intact fungal endophytes, were sunk into pits outside each of the OTCs. Second, 140 seeds of *P. annua* growing in the study site, 70 with intact fungal endophytes (E^+^) and other 70 with reduced endophytes (E^-^), were sown at 1–2 cm depth into pits of each treatment indicated above (inside and outside OTCs). This is the approximate depth at which *P. annua* seeds are found at the study site (pers. obs.). Survival was recorded once per month for four months, and the germination was assessed as the number of seedlings that emerged being recorded every week. Inside and outside each OTC, pits filled only with sterilized and unsterilized soil was placed to assess the potential effect of plant emergence due to seed rain.

### Open top chambers design

Seven OTCs were installed on the Ecology Glacier forefield in January 2017 to simulate the passive warming expected under projected climate scenarios for Maritime Antarctica. Each OTC was constructed from 4 mm transparent acrylic sheeting and enclosed a basal area of 692 cm². To prevent excessive heat accumulation, 20 circular ventilation holes (Ø = 1.5 cm) were drilled into the chamber walls and the uppermost approximately 1–2 cm of soil was removed from beneath the wall edges, excluding corners, to allow for lateral airflow. OTCs were placed 100 m from the glacier front and separated from each other by 2 m (**Figure 1**). This passive warming system has been previously validated at this study site and shown to increase air temperature by 1.8 ± 1.0 °C and decrease soil water potential by an average of 2.2 kPa in chambered soils compared with unchambered control soils (for details, see Acuña-Rodríguez et al., 2024). Unfortunately, some OTC were misplaced due to adverse weather conditions (mainly wind and snow displacement), reducing the replicates to 5 per treatment combination (OTC × E).

### Removal of endophytes from *P. annua* tillers and seeds

To obtain endophyte-reduced tillers (E^−^), plants were treated for five days with the systemic fungicide Benlate^®^ (benomyl; DuPont) at 0.5 g L^-1^, while control tillers (E^+^) received equivalent volumes of sterile water. Treatment efficacy was verified in three randomly selected plants per group using two complementary methods: (i) root cross-sections stained with trypan blue in acidified glycerol were examined at 400× magnification (Motic BA410) for the presence of fungal hyphae; and (ii) root segments were plated on potato dextrose agar (PDA; Difco) supplemented with chloramphenicol (100 g mL^-1^) and incubated at 18 °C for two weeks. Tillers were classified as endophyte-reduced only when no fungal outgrowth was detected on culture plates. Conversely, E^+^ tillers consistently showed fungal hyphae in stained cross-sections and yielded fungal colonies on culture plates, confirming intact endophyte communities in the control group.

For seed endophyte removal, seed surfaces were first disinfected by immersion in 1% sodium hypochlorite for 30 seconds, followed by three rinses in sterile distilled water. Seeds were then submerged in a Benlate^®^ solution (2 g L^-1^) for 30 minutes at room temperature, and this treatment was repeated on three consecutive days using the same seed batch. Endophyte reduction was subsequently confirmed by staining cross-sections of five seeds with aniline blue to visualize fungal hyphae, and by plating seed fragments on PDA with chloramphenicol (100 µg mL^-^¹) at 18 °C. Seeds were considered fungal endophyte-reduced and suitable for the experiment only in the absence of any fungal colony growth. This fungicide has been shown to effectively reduce endophyte loads in *P. annua* without causing phytotoxic effects on Antarctic plants under comparable experimental conditions (Barrera et al., 2020; Hereme et al., 2020). E^+^ seeds showed fungal hyphae in aniline blue-stained cross-sections and yielded fungal colonies on PDA, confirming the presence of fungal endophytes in the control seed batch.

At the end of the experiment, all *P. annua* individuals, together with all plant debris and reproductive structures, were incinerated in a metal drum. Additionally, the paper pots containing any non-germinated *P. annua* seeds were also incinerated following the same procedure. These procedures effectively eliminated the risk of seed dispersal and prevented the germination or establishment of new *P. annua* individuals within the Antarctic environment.

### Gene expression

The relative expression of *HSP* and *LEA1* genes was quantified in leaf tissue harvested from five *P. annua* plants per treatment group after three weeks of growth. Total RNA was extracted using a modified sodium perchlorate method (5 M sodium perchlorate, 300 mM Tris-HCl pH 8.0, 1% v/v SDS, 2% v/v PEG 20,000, 8.5% w/v PVPP, 3% v/v 2-mercaptoethanol) following a previous protocol (Davies and Robinson, 1996). Residual genomic DNA was removed with Turbo DNase (Ambion), and first-strand cDNA was synthesized from 2 µg of total RNA using the Maxima H Minus First Strand cDNA Synthesis Kit (Thermo Fisher Scientific). Quantitative real-time PCR was performed on a Mx3000P system (Agilent) using Maxima SYBR Green PCR Master Mix (Thermo Fisher Scientific). Gene-specific primers were designed with NCBI Primer Blast against the *P. annua* transcriptome assembly GCZY01014682.1 (Chen et al., 2016): *HSP* (Fw: 5′-CTTGCTGAGGCCGATGAGTTCG-3′; Rv: 5′-CTCATCCATGCCACCAGCCATG-3′) and *LEA1* (Fw: 5’ TGAGCGTGATGAGGAAGTCG 3’ - Rv: 5’ CACCAACCCATACTCCCACC 3’). Each 20 µL reaction contained 2 µL diluted cDNA (50 ng), 10 µL SYBR Green master mix, 0.8 µL of each primer (10 µM), and 6.4 µL nuclease-free water. Cycling conditions were 95 °C for 10 min; 40 cycles of 95 °C for 15 s, 60 °C for 15 s, 72 °C for 20 s; followed by a dissociation stage (95 °C for 15 s, 54 °C for 15 s, 95 °C for 30 s). All reactions were run in triplicate. Relative expression was calculated using the 2^−ΔΔCT^ method (Livak and Schmittgen, 2001), with elongation factor 1α (*EF1α*) as the reference gene, as described in (LaForest et al., 2021).

### Statistical analyses

All analyses were performed using R v.4.5.3 (R Core Team, 2026). Data distribution and residual structure were explored visually through Q–Q plots and histograms, and normality and homoscedasticity were formally assessed using the Shapiro–Wilk test and Levene’s test, respectively, as implemented in the ‘*car’* package (Fox and Weisberg, 2019). Differences in germination, survival and relative gene expression (*HSP* and *LEA1*) in *P. annua* plants were assessed separately for each response variable using generalized linear models (GLMs) fitted with the ‘*glm’* function. A quasibinomial distribution with a ‘*logit*’ link function was used for germination and survival data, which were expressed as proportions (data shown in percentage in figures). For relative gene expression of both *HSP* and *LEA1* genes, the best-fitting error distribution was selected by comparing Gaussian and Gamma families using the Akaike Information Criterion (AIC), with the Gamma distribution with a ‘*log*’ link function providing the better fit in both cases. All models included the fixed effects of warming treatment (OTC), endophyte presence (E), and their interaction (OTC × E). In the experimental design, OTC identity constituted the primary experimental unit for the warming treatment, with individual plants and seeds nested within chambers. To account for this structure, OTC was treated as a fixed factor in the model rather than a random grouping variable, as the number of chambers (n = 5 after weather-related losses) precluded reliable estimation of random-effect variance. Treatment-level means per OTC were used as the analytical unit for the warming factor, and the degrees of freedom were assigned accordingly. Model fit and residual adequacy were evaluated through simulation-based scaled residuals using the ‘*DHARMa’* package (Hartig, 2026), which provides uniformity, dispersion and outlier diagnostics valid across GLM families. *Post-hoc* pairwise comparisons were conducted on estimated marginal means using the ‘*emmeans’* package (Lenth, 2026), with *p*-values adjusted using the Šidák correction. Figures were produced using ‘*ggplot2’* (Wickham, 2016) and ‘*patchwork’* (Pedersen, 2025).

## Results

### Germination and survival

**Figure 2:**
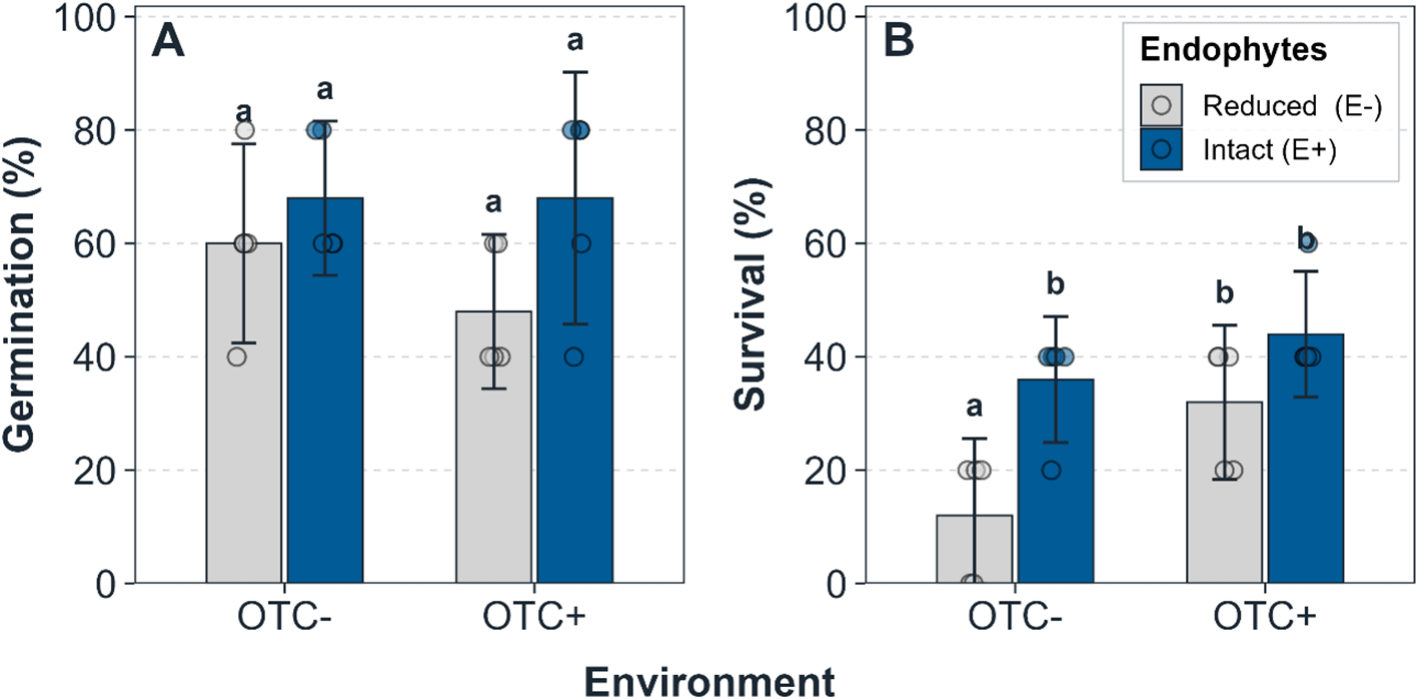
Germination **(A)** and survival **(B)** of *Poa annua* seedlings under open top chamber (OTC^−^, ambient temperature; OTC^+^, experimentally warmed) and endophytes treatments (E^−^, endophytes reduced; E^+^, endophytes intact). Bars represent treatment means with confidence intervals (95% CI). Different lowercase letters above bars denote significant differences among treatment groups based on Šidák-adjusted pairwise comparisons of GLM estimated marginal means (*p* < 0.05). *n* = 5 per treatment combination.

Seed germination of *P. annua* did not differ significantly among treatment groups, as the model revealed no significant effect of warming treatment, endophyte presence, or their interaction (**Supplementary Table 1**). Although a numerical decrease of 21% was observed in E^−^ plants relative to E^+^ plants inside OTC^+^, this difference did not reach statistical significance (*Post-hoc* comparison: *p* > 0.05; **Figure 2A**).

Seedling survival, in contrast, was significantly influenced by both warming (OTC: *z* = 3.04, *p* = 0.008) and endophyte presence (E: *z* = 3.51, *p* = 0.003), with no significant interaction between these factors (*p* > 0.05, **Supplementary Table 1**; **Figure 2B**). Plants in the OTC^−^ E^−^ treatment exhibited the lowest survival, which differed significantly from OTC^+^ E^−^ (*p* = 0.014), OTC^−^ E^+^ (*p* = 0.003), and OTC^+^ E^+^ (*p* < 0.001). No significant differences were detected among the remaining treatment pairs (*p* > 0.05).

### HSP and LEA1 gene expression

**Figure 3:**
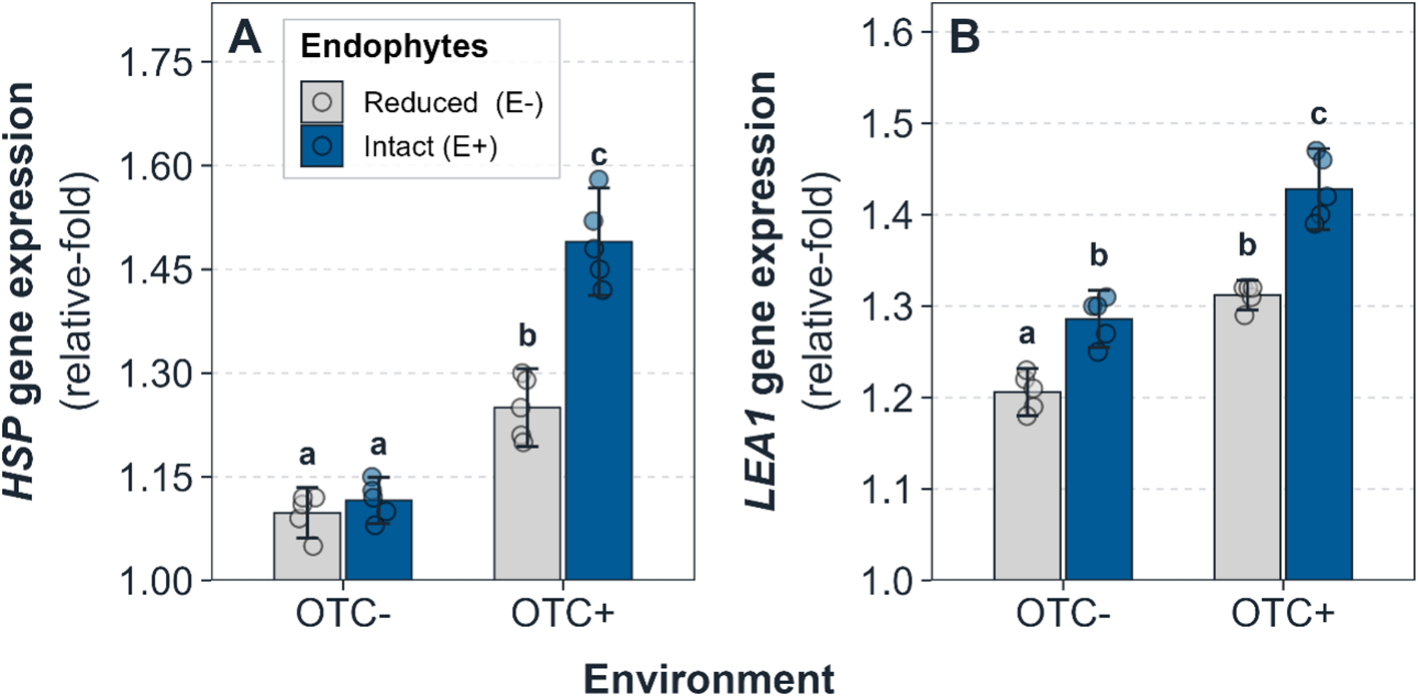
Relative gene expression of the heat shock protein gene *HSP* **(A)** and the late embryogenesis abundant gene *LEA1* **(B)** in *Poa annua* under open top chamber (OTC^−^, ambient temperature; OTC^+^, experimentally warmed) and endophytes treatments (E^−^, endophytes reduced; E^+^, endophytes intact). Bars represent treatment means with confidence intervals (95% CI). Different lowercase letters above bars denote significant differences among treatment groups based on Šidák-adjusted pairwise comparisons of GLM estimated marginal means (*p* < 0.05). Expression values are presented as relative-fold change. *n* = 5 per treatment combination.

*HSP* gene expression was significantly affected by warming (*t* = 6.20, *p* < 0.001) and by the OTC × E interaction (*t* = 5.39, *p* < 0.001), but not by endophyte presence alone (*p* = 0.448; **Supplementary Table 1**; **Figure 3A**). *Post-hoc* comparisons revealed that plants under OTC_−_ conditions, regardless of endophyte status, showed the lowest *HSP* expression and did not differ from each other (*p* > 0.05). Under warming, *HSP* expression increased markedly, with OTC^+^ E^−^ plants showing intermediate expression and OTC^+^ E^+^ plants exhibiting the highest values, both differing significantly from all other groups (*p* < 0.001).

*LEA1* gene expression was significantly affected by both warming (OTC: *t* = 7.12, *p* < 0.001) and endophyte presence (E: *t* = 5.43, *p* < 0.001), with no significant interaction (**Supplementary Table 1**; **Figure 3B**). The lowest expression was observed in OTC^−^ E^−^ plants, which differed significantly from all other treatments (*p* < 0.001). Plants in OTC^−^ E^+^ and OTC^+^ E^−^ showed comparable intermediate expression levels (*p* > 0.05), while OTC^+^ E^+^ plants exhibited the highest *LEA1* expression, differing significantly from all remaining groups (*p* < 0.001).

## Discussion

Fungal endophytes are known to enhance the colonization success of invasive plants by improving stress tolerance and plant performance (Oses-Pedraza et al., 2020; Ballesteros et al., 2022; Molina-Montenegro et al., 2023, Molina-Montenegro et al., 2026), although their importance may depend strongly on environmental conditions (Hereme et al., 2020). However, while previous studies indicated seed-borne fungal endophytes from *Poa annua* enhances germination (Ballesteros et al., 2022; Molina-Montenegro et al., 2023), our findings showed that neither the presence of endophytes nor experimental warming (OTC) significantly affected germination. The most likely explanation lies in the moisture conditions experienced by germinating seeds at our study site. The Ecology Glacier forefield adjacent to H. Arctowski Station is a characteristically moist coastal environment (Plenzler et al., 2026), in contrast to the more water-limited conditions reported in previous experiments (Ballesteros et al., 2022; Molina-Montenegro et al., 2023). The benefit of endophytes to early germination tends to be most pronounced under abiotic stress (Rodriguez et al., 2009), and when moisture is not limiting, endophyte-mediated facilitation may become negligible. This interpretation is consistent with the broader principle that plant–microbe mutualisms express their positive effects most clearly under stressful conditions and shift towards neutrality as abiotic pressure is relaxed (Torres-Díaz et al., 2016; Hereme et al., 2020). In this context, water availability is the main factor controlling germination. Since our site provides plenty of moisture (Plenzler et al., 2026), seeds reach their needs easily on their own. This makes the help from endophytes unnecessary, explaining why their effect disappears and results in the neutral trend observed.

On the other hand, seedling survival responded to both warming and endophyte presence. Under “current” conditions (OTC^−^), plants without endophytes showed the lowest survival (12%), while plants with intact endophytes nearly tripled this value (36%). This confirms that seed-borne microorganisms play a meaningful role in *P. annua* establishment under present-day Antarctic conditions. However, warming alone (OTC^+^ E^−^) produced a comparable improvement in survival (32%), and the OTC × E interaction was not significant, indicating that the benefits of endophyte presence and warming operate independently rather than synergistically on plant persistence. This suggests that as Antarctic temperatures continue to rise (Bromwich et al., 2026), warming alone may provide the same survival benefits as endophytes, potentially reducing the plant’s reliance on these symbionts during establishment. Previous work on Antarctic native plants documented a similar pattern where fungal endophytes improved ecophysiological performance under current stress but became functionally neutral under warmer, wetter projected conditions (Torres-Díaz et al., 2016; Hereme et al., 2020). If *P. annua* follows the same trajectory, its invasive spread could accelerate, not because endophytes confer greater advantage, but because the plant becomes capable of establishing without them across an expanding ice-free landscape (Convey and Peck, 2019).

Regarding gene expression, under ambient conditions, endophyte presence had no detectable effect on *HSP* transcription, while warming alone produced a moderate increase. Critically, the combination of warming and endophytes resulted in a markedly higher *HSP* expression than any other treatment group, revealing that endophytes amplify the heat-stress transcriptional response specifically when the plant is thermally challenged. *HSP*s are molecular chaperones whose primary role is to prevent irreversible denaturation of proteins and to assist in the refolding of misfolded complexes under heat stress (Kotak et al., 2007; Waters, 2012); their up-regulation is a hallmark of acquired thermotolerance in plants (Sung et al., 2003). The endophyte-driven amplification of this response is consistent with earlier experimental evidence that fungal endophytes can enhance molecular protection networks associated with thermal tolerance in host plants (Redman et al., 2002), though whether this transcriptional priming translates into measurable thermotolerance in *P. annua* remains to be directly tested. In our results, the absence of an endophyte effect on *HSP* expression under ambient conditions, together with its strong increase under warming, suggests that endophytes enhance heat-stress responses only when plants are exposed to higher temperatures, likely requiring an environmental stress threshold to activate the response. For *P. annua*, this conditional response may be especially relevant as the mean temperature is increasing in Maritime Antarctica (Wang et al., 2025).

Unlike *HSP*, the expression of the *LEA1* gene showed additive and independent effects of warming and endophyte presence, without a significant interaction. LEA proteins protect cells from desiccation and osmotic damage by stabilizing membranes and macromolecules during water deficit, and are induced by both heat and drought stress (Close, 1996; Hundertmark and Hincha, 2008). The fact that endophytes alone, even under ambient temperatures, were sufficient to up-regulate *LEA1* expression suggests that these microorganisms may prime *P. annua* for dehydration stress even before thermal conditions change. The lack of a synergistic interaction between factors suggests that, although OTCs typically increase evapotranspiration, the proximity to the glacial front may provide sufficient soil moisture to buffer severe thermal-induced water deficit (Acuña-Rodríguez et al., 2024). This is ecologically relevant because soil moisture in Antarctica can fluctuate substantially between spatiotemporal scales (Hrbáček et al., 2023), and newly exposed substrates following glacial retreat are often characterized by intermittent drying (Acuña-Rodríguez et al., 2024). Together, the *HSP* and *LEA1* results indicate that fungal endophytes act as molecular amplifiers of the stress-response gene network in *P. annua*, upregulating transcriptional pathways associated with both thermal and hydric stress protection. However, whether this enhanced gene expression translates into expanded physiological tolerance will require direct functional validation.

Taken together, our results suggest that the ecological role of seed-borne fungal endophytes in *Poa annua* is context dependent and changes with environmental conditions. Under current Antarctic conditions, endophytes were associated with improved seedling survival, supporting their potential role in facilitating establishment when environmental constraints remain strong. Nonetheless, under experimental warming, their most evident influence emerged at the molecular level, where endophyte-associated plants exhibited enhanced expression of stress-response genes. This pattern indicates that warming may alter how plant–endophyte interactions are expressed rather than simply increasing or decreasing their importance, shifting the most evident effects from demographic performance (germination and survival) to molecular responses (gene expression). Although the functional consequences of these transcriptional changes remain to be determined, the enhanced expression of *HSP70* and *LEA1* under warming is consistent with the possibility that fungal endophytes contribute to the activation of protective stress-response pathways (Redman et al., 2002; Acuña-Rodríguez et al., 2024).

As climate warming continues to expand ice-free areas across Maritime Antarctica —projected to increase by up to 25% by the end of the XXI century, with a threefold increase specifically in the Antarctic Peninsula (Lee et al., 2017)— the availability of suitable habitat for *P. annua* is expected to grow substantially, further compounding the threat that non-native species pose to Antarctic terrestrial biodiversity (Duffy and Lee, 2019). This expanding suitability is compounded by the fact that populations from multiple geographic origins show comparable capacity to establish under Antarctic conditions, meaning that invasion risk is not constrained to a single propagule source (Rudak et al., 2026). Our results suggest that under warmer conditions, *P. annua* may be capable of colonizing this new terrain without requiring endophyte-mediated facilitation for establishment, making understanding how fungal endophytes influence both early survival and molecular stress responses critical for anticipating the future dynamics of biological invasions in one of the world’s most rapidly changing ecosystems.

## Data Availability

The datasets presented in this study can be found in online repositories. The names of the repository/repositories and accession number(s) can be found below: https://doi.org/10.6084/m9.figshare.32118787.

## References

[B1] Acuña-Rodríguez I. S. Newsham K. K. Convey P. Biersma E. M. Ballesteros G. I. Torres-Díaz C. . (2024). The role of the soil microbiome in the colonisation of glacier forefields by Antarctic pearlwort (Colobanthus quitensis) under current and future climate change scenarios. Soil Biol. Biochem. 188, 109249. doi: 10.1016/j.soilbio.2023.109249 38826717

[B2] Ballesteros G. I. Acuña-Rodríguez I. S. Barrera A. Gundel P. E. Newsham K. K. Molina-Montenegro M. A. . (2022). Seed fungal endophytes promote the establishment of invasive Poa annua in maritime Antarctica. Plant Ecolog. Divers. 15, 199–212. doi: 10.1080/17550874.2022.2145579 37339054

[B3] Barrera A. Hereme R. Ruiz-Lara S. Larrondo L. F. Gundel P. E. Pollmann S. . (2020). Fungal endophytes enhance the photoprotective mechanisms and photochemical efficiency in the Antarctic Colobanthus quitensis (Kunth) Bartl. exposed to UV-B radiation. Front. Ecol. Evol. 8. doi: 10.3389/fevo.2020.00122

[B4] Bokhorst S. Huiskes A. Aerts R. Convey P. Cooper E. J. Dalen L. . (2013). Variable temperature effects of open top chambers at polar and alpine sites explained by irradiance and snow depth. Global Change Biol. 19, 64–74. doi: 10.1111/gcb.12028 23504721

[B5] Bromwich D. H. Zou X. Wang S.-H. (2026). Interior Antarctica is undergoing marked climate change. Commun. Earth Environ. 7, 389. doi: 10.1038/s43247-026-03384-4 37880705

[B6] Chen S. McElroy J. S. Dane F. Goertzen L. R. (2016). Transcriptome assembly and comparison of an allotetraploid weed species, annual bluegrass, with its two diploid progenitor species, Poa supina Schrad and Poa infirma Kunth. Plant Genome 9, plantgenome2015.2006.0050. doi: 10.3835/plantgenome2015.06.0050 27898765

[B7] Chown S. L. Huiskes A. H. L. Gremmen N. J. M. Lee J. E. Terauds A. Crosbie K. . (2012). Continent-wide risk assessment for the establishment of nonindigenous species in Antarctica. Proc. Natl. Acad. Sci. 109, 4938–4943. doi: 10.1073/pnas.1119787109 22393003 PMC3323995

[B8] Chwedorzewska K. J. Giełwanowska I. Olech M. Molina-Montenegro M. A. Wódkiewicz M. Galera H. (2015). Poa annua L. in the maritime Antarctic: an overview. Polar Rec. 51, 637–643. doi: 10.1017/S0032247414000916 41292463

[B9] Close T. J. (1996). Dehydrins: emergence of a biochemical role of a family of plant dehydration proteins. Physiol. Plant 97, 795–803. doi: 10.1111/j.1399-3054.1996.tb00546.x 40046247

[B10] Convey P. Peck L. S. (2019). Antarctic environmental change and biological responses. Sci. Adv. 5, eaaz0888. doi: 10.1126/sciadv.aaz0888 31807713 PMC6881164

[B11] Davies C. Robinson S. P. (1996). Sugar accumulation in grape berries (cloning of two putative vacuolar invertase cDNAs and their expression in grapevine tissues). Plant Physiol. 111, 275–283. doi: 10.1104/pp.111.1.275 8685267 PMC157835

[B12] Duffy G. A. Lee J. R. (2019). Ice-free area expansion compounds the non-native species threat to Antarctic terrestrial biodiversity. Biol. Conserv. 232, 253–257. doi: 10.1016/j.biocon.2019.02.014 38826717

[B13] Fox J. Weisberg S. (2019). An R Companion to Applied Regression, Third Edition. Thousand Oaks, CA: Sage (SAGE Publications)

[B14] Frenot Y. Chown S. L. Whinam J. Selkirk P. M. Convey P. Skotnicki M. . (2005). Biological invasions in the Antarctic: extent, impacts and implications. Biol. Rev. 80, 45–72. doi: 10.1017/S1464793104006542 15727038

[B15] González-Herrero S. Barriopedro D. Trigo R. M. López-Bustins J. A. Oliva M. (2022). Climate warming amplified the 2020 record-breaking heatwave in the Antarctic Peninsula. Commun. Earth Environ. 3, 122. doi: 10.1038/s43247-022-00450-5 37880705

[B16] Hartig F. (2026). Dharma: Residual Diagnostics for Hierarchical (Multi-Level / Mixed) Regression Models. R Package Version 0.5.0. Available online at: https://CRAN.R-project.org/package=DHARMa

[B17] Hereme R. Morales-Navarro S. Ballesteros G. Barrera A. Ramos P. Gundel P. E. . (2020). Fungal endophytes exert positive effects on Colobanthus quitensis under water stress but neutral under a projected climate change scenario in Antarctica. Front. Microbiol. 11. doi: 10.3389/fmicb.2020.00264 32184767 PMC7058981

[B18] Hrbáček F. Uxa T. Láska K. (2023). Variability of soil moisture on three sites in the Northern Antarctic Peninsula in 2022/23. Czech. Polar Rep. 13, 10–23. doi: 10.5817/CPR2023-1-2

[B19] Hundertmark M. Hincha D. K. (2008). LEA (Late Embryogenesis Abundant) proteins and their encoding genes in Arabidopsis thaliana. BMC Genomics 9, 118. doi: 10.1186/1471-2164-9-118 18318901 PMC2292704

[B20] Kotak S. Larkindale J. Lee U. von Koskull-Döring P. Vierling E. Scharf K.-D. (2007). Complexity of the heat stress response in plants. Curr. Opin. Plant Biol. 10, 310–316. doi: 10.1016/j.pbi.2007.04.011 17482504

[B21] LaForest M. Soufiane B. Patterson E. L. Vargas J. J. Boggess S. L. Houston L. C. . (2021). Differential expression of genes associated with non-target site resistance in Poa annua with target site resistance to acetolactate synthase inhibitors. Pest. Manage. Sci. 77, 4993–5000. doi: 10.1002/ps.6541 34218510 PMC8518846

[B22] Lee J. R. Raymond B. Bracegirdle T. J. Chadès I. Fuller R. A. Shaw J. D. . (2017). Climate change drives expansion of Antarctic ice-free habitat. Nature 547, 49–54. doi: 10.1038/nature22996 28658207

[B23] Lenth R. (2026). Emmeans: Estimated Marginal Means, Aka Least-Squares Means. R Package Version 2.0.3. Available online at: https://CRAN.R-project.org/package=emmeans

[B24] Livak K. J. Schmittgen T. D. (2001). Analysis of relative gene expression data using real-time quantitative PCR and the 2-ΔΔCT method. Methods 25, 402–408. doi: 10.1006/meth.2001.1262 11846609

[B25] Molina-Montenegro M. A. Acuña-Rodríguez I. S. Atala C. Ballesteros G. I. Carrasco-Urra F. Castro-Nallar E. . (2026). The endomicrobiome and weed invasiveness in Mediterranean ecosystems worldwide. Nat. Commun. 17, 3063. doi: 10.1038/s41467-026-68826-1 41730892 PMC13039264

[B26] Molina-Montenegro M. A. Ballesteros G. I. Acuña-Rodríguez I. S. Pertierra L. R. Greve M. Richardson D. M. . (2023). The “Trojan horse” strategy: seed fungal endophyte symbiosis helps to explain the invasion success of the grass, Poa annua, in Maritime Antarctica. Divers. Distrib. 29, 1432–1444. doi: 10.1111/ddi.13768 40046247

[B27] Molina-Montenegro M. A. Carrasco-Urra F. Rodrigo C. Convey P. Valladares F. Gianoli E. (2012). Occurrence of the non-native annual bluegrass on the Antarctic mainland and its negative effects on native plants. Conserv. Biol. 26, 717–723. doi: 10.1111/j.1523-1739.2012.01865.x 22624790

[B28] Newsham K. K. Misiak M. Goodall-Copestake W. P. Dahl M. S. Boddy L. Hopkins D. W. . (2022). Experimental warming increases fungal alpha diversity in an oligotrophic maritime Antarctic soil. Front. Microbiol. 13, 1050372. doi: 10.3389/fmicb.2022.1050372 36439821 PMC9684652

[B29] Oses-Pedraza R. Torres-Díaz C. Lavín P. Retamales-Molina P. Atala C. Gallardo-Cerda J. . (2020). Root endophytic Penicillium promotes growth of Antarctic vascular plants by enhancing nitrogen mineralization. Extremophiles 24, 721–732. doi: 10.1007/s00792-020-01189-7 32699913

[B30] Pedersen T. (2025). Patchwork: The Composer of Plots. R Package Version 1.3.2.9000. Available online at: https://CRAN.R-project.org/package=patchwork

[B31] Plenzler J. Piotrowicz K. Owczarek M. (2026). Biometeorological conditions at Polish Antarctic Station (King George Island, West Antarctica) according to Universal Thermal Climate Index 2013–2023. Int. J. Biometeorol. 70, 69. doi: 10.1007/s00484-025-03099-9 41718770 PMC12923444

[B32] Pyšek P. Hulme P. E. Simberloff D. Bacher S. Blackburn T. M. Carlton J. T. . (2020). Scientists' warning on invasive alien species. Biol. Rev. 95, 1511–1534. doi: 10.1111/brv.12627 32588508 PMC7687187

[B33] R Core Team (2026). R: A Language and Environment for Statistical Computing. R Foundation for Statistical Computing (Vienna, Austria: R Foundation for Statistical Computing).

[B34] Redman R. S. Sheehan K. B. Stout R. G. Rodriguez R. J. Henson J. M. (2002). Thermotolerance generated by plant/fungal symbiosis. Science 298, 1581–1581. doi: 10.1126/science.1078055 12446900

[B35] Rodriguez R. J. White J. F. Arnold A. E. Redman R. S. (2009). Fungal endophytes: diversity and functional roles. New Phytol. 182, 314–330. doi: 10.1111/j.1469-8137.2009.02773.x 19236579

[B36] Rudak A. Galera H. Wódkiewicz M. (2026). Antarctica may be vulnerable to invasion by Poa annua from a range of populations around the globe. J. Appl. Ecol. 63, e70250. doi: 10.1111/1365-2664.70250 40046247

[B37] Rudak A. Galera H. Znoj A. Chwedorzewska K. J. Wodkiewicz M. (2018). Seed germination and invasion success of Poa annua L. in Antarctica. Acta Societatis. Botanicorum. Poloniae. 87, 1c+. doi: 10.5586/asbp.3606

[B38] Sung D.-Y. Kaplan F. Lee K.-J. Guy C. L. (2003). Acquired tolerance to temperature extremes. Trends Plant Sci. 8, 179–187. doi: 10.1016/S1360-1385(03)00047-5 12711230

[B39] Torres-Díaz C. Gallardo-Cerda J. Lavin P. Oses R. Carrasco-Urra F. Atala C. . (2016). Biological interactions and simulated climate change modulates the ecophysiological performance of Colobanthus quitensis in the Antarctic ecosystem. PloS One 11, e0164844. doi: 10.1371/journal.pone.0164844 27776181 PMC5077106

[B40] Wang S. Li G.-C. Zhang Z.-H. Zhang W.-Q. Wang X. Chen D. . (2025). Recent warming trends in Antarctica revealed by multiple reanalysis. Adv. Clim. Change Res. 16, 447–459. doi: 10.1016/j.accre.2025.03.003 38826717

[B41] Waters E. R. (2012). The evolution, function, structure, and expression of the plant sHSPs. J. Exp. Bot. 64, 391–403. doi: 10.1093/jxb/ers355 23255280

[B42] Wickham H. (2016). Ggplot2: Elegant Graphics for Data Analysis (New York: Springer-Verlag).

