# Supplementary material for: Warming reduces fungal endophyte dependency for establishment but amplifies stress-response gene expression in the Antarctic invader Poa annua
Source: Front Fungal Biol. 2026 Jul 8;7:1868127. doi: 10.3389/ffunb.2026.1868127 (PMC13388306; doi:10.3389/ffunb.2026.1868127)
Supplement: Supplementary file 1 [file Table1.docx]

**Suplementary material**

**Table S1.** Results of generalized linear models (GLMs) assessing the effects of warming treatment (OTC), endophyte presence (E), and their interaction (OTC × E) on germination, survival, *HSP* gene expression and *LEA1* gene expression in *Poa annua*. Germination and survival were modelled using a quasibinomial distribution with a logit link function; *HSP* and *LEA1* expression were modelled using a Gamma distribution with a log link function. For quasibinomial family analyses the *z*-value is reported. For Gamma family analyses the *t*-value is reported. Significant differences in factors are denoted in black bold for *t*/*z*-values and red bold for *P*-values. Lower and upper 95% confidence intervals (CI) and standard deviations (SD) are also reported for each estimate.

| **Response variable** | **Term** | **Estimate** |  | **Lower CI** | **Upper  CI** | **SD** | **t / z** | ***P*-value** |
| --- | --- | --- | --- | --- | --- | --- | --- | --- |
| **Germination (%)** | Intercept | 0.405 |  | -0.105 | 0.934 | 0.264 | 1.538 | 0.144 |
|  | Environment (OTC) | −0.486 |  | -1.217 | 0.235 | 0.369 | −1.315 | 0.207 |
|  | Endophytes presence (E) | 0.348 |  | -0.399 | 1.105 | 0.382 | 0.911 | 0.376 |
|  | OTC × E | 0.486 |  | -0.569 | 1.545 | 0.538 | 0.902 | 0.380 |
| **Survival (%)** | Intercept | −1.992 |  | -2.710 | -1.385 | 0.334 | **−5.961** | **< 0.001** |
|  | Environment (OTC) | 1.239 |  | 0.467 | 2.077 | 0.407 | **3.041** | **0.008** |
|  | Endophytes presence (E) | 1.417 |  | 0.654 | 2.250 | 0.404 | **3.511** | **0.003** |
|  | OTC × E | −0.904 |  | -1.938 | 0.089 | 0.515 | −1.757 | 0.098 |
| ***HSP* expression** | Intercept | 0.093 |  | 0.065 | 0.123 | 0.015 | **6.320** | **< 0.001** |
|  | Environment (OTC) | 0.130 |  | 0.089 | 0.171 | 0.021 | **6.198** | **< 0.001** |
|  | Endophytes presence (E) | 0.016 |  | -0.025 | 0.057 | 0.021 | 0.777 | 0.448 |
|  | OTC × E | 0.159 |  | 0.101 | 0.217 | 0.030 | **5.387** | **< 0.001** |
| ***LEA1* expression** | Intercept | 0.187 |  | 0.171 | 0.204 | 0.008 | **22.403** | **< 0.001** |
|  | Environment (OTC) | 0.084 |  | 0.061 | 0.107 | 0.012 | **7.125** | **< 0.001** |
|  | Endophytes presence (E) | 0.064 |  | 0.041 | 0.087 | 0.012 | **5.432** | **< 0.001** |
|  | OTC × E | 0.020 |  | -0.012 | 0.053 | 0.017 | 1.226 | 0.238 |
